# Upregulation of Human ST8Sia VI (α2,8-Sialyltransferase) Gene Expression by Physcion in SK-N-BE(2)-C Human Neuroblastoma Cells

**DOI:** 10.3390/ijms17081246

**Published:** 2016-08-02

**Authors:** Hyun-Kyoung Yoon, Hyun-Kyu An, Min Jung Ko, Kyoung-Sook Kim, Seo-Won Mun, Dong-Hyun Kim, Cheol Min Kim, Cheorl-Ho Kim, Young Whan Choi, Young-Choon Lee

**Affiliations:** 1 Department of Medicinal Biotechnology, College of Health Sciences, Dong-A university, Busan 49315, Korea; gusrud073@naver.com (H.-K.Y.); beastne@nate.com (H.-K.A.); kskim@dau.ac.kr (K.-S.K.); ss11033@hanmail.net (S.-W.M.); mose79@dau.ac.kr (D.-H.K.); 2 Department of Horticultural Bioscience, Pusan National University, Miryang 50463, Korea; komj99@pusan.ac.kr; 3 Research Center for Anti-Aging Technology Development, Pusan National University, Busan 46241, Korea; kimcm@pusan.ac.kr; 4 Molecular and Cellular Glycobiology Unit, Department of Biological Sciences, SungKyunKwan University, Kyunggi-Do 16419, Korea; chkimbio@skku.edu

**Keywords:** physcion, hST8Sia VI, SK-N-BE(2)-C, transcription factor Pax-5, signal pathway

## Abstract

In this research, we firstly demonstrated that physcion, an anthraquinone derivative, specifically increased the expression of the human α2,8-sialyltransferase (hST8Sia VI) gene in SK-N-BE(2)-C human neuroblastoma cells. To establish the mechanism responsible for the up-regulation of hST8Sia VI gene expression in physcion-treated SK-N-BE(2)-C cells, the putative promoter region of the hST8Sia VI gene was functionally characterized. Promoter analysis with serially truncated fragments of the 5′-flanking region showed that the region between −320 and −240 is crucial for physcion-induced transcription of hST8Sia VI in SK-N-BE(2)-C cells. Putative binding sites for transcription factors Pax-5 and NF-Y are located at this region. The Pax-5 binding site at −262 to −256 was essential for the expression of the hST8Sia VI gene by physcion in SK-N-BE(2)-C cells. Moreover, the transcription of hST8Sia VI induced by physcion in SK-N-BE(2)-C cells was inhibited by extracellular signal-regulated protein kinase (ERK) inhibitor U0126 and p38 mitogen-activated protein kinase (MAPK) inhibitor SB203580, but not c-Jun *N*-terminal kinase (JNK) inhibitor SP600125. These results suggest that physcion upregulates hST8Sia VI gene expression via ERK and p38 MAPK pathways in SK-N-BE(2)-C cells.

## 1. Introduction

Sialic acid (NeuAc) residues play crucial roles in diverse cellular events, including intercellular adhesion, cell differentiation, microbial attachment, and malignant transformation [1]. The sialylated glycans are formed by a family of sialyltransferases that catalyze the terminal addition of sialic acid to glycan chains [2]. Mammalian sialyltransferases characterized so far exhibit distinct specificities for acceptor substrate for glycoproteins and glycolipids and their gene expressions show marked tissue- and cell type-specific patterns, which is closely connected with cell type-specific glycan structures [2,3,4,5,6]. Because cell type-specific gene expression is generally known to be regulated on the transcriptional level, dissecting the molecular mechanisms for cell type-specific expression of sialyltransferase genes may be essential for understanding cell type-specific sialylation of glycoproteins and glycosphingolipids.

Recently we studied the transcriptional regulation mechanism of human sialyltransferase genes that are specifically expressed at high levels in cell lines treated with natural compounds [7,8,9,10,11]. In the current study, we investigated the effect of physcion isolated from the *Polygonum multiflorum* roots on the human sialyltransferase gene expression in SK-N-BE(2)-C human neuroblastoma cells.

Physcion is an anthraquinone derivative isolated from rhubarb, a Chinese herb medicine [12] and the marine-derived fungus *Microsporum* sp. [13]. Previous studies have demonstrated that physcion has various pharmacological activities such as hepatoprotective [14], anti-microbial [15], and anti-inflammatory effects [16]. In addition, recent studies have also demonstrated that physcion has anti-cancer effects through proliferation inhibition and apoptosis induction in cervical [13], breast [17], and colorectal cancer cells [18]. It was also recently reported that physcion has an anti-metastatic effect against human colorectal cancer SW620 cells by repressing the transcription factor SOX2 [19]. However, the effects of physcion on the expression of various genes, including sialyltransferase genes, and its underlying molecular mechanisms have never been investigated.

Therefore, this work was undertaken to assess the effects of physcion on the gene expression of human sialyltransferases and the underlying mechanisms. In the present study, we discovered for the first time the specific increase of the human α2,8-sialyltransferase (hST8Sia VI) gene expression by physcion in human neuroblastoma SK-N-BE(2)-C cells and the molecular basis of physcion-induced hST8Sia VI gene expression was also investigated.

## 2. Results

### 2.1. Isolation and Identification of Physcion

**Figure 1:**
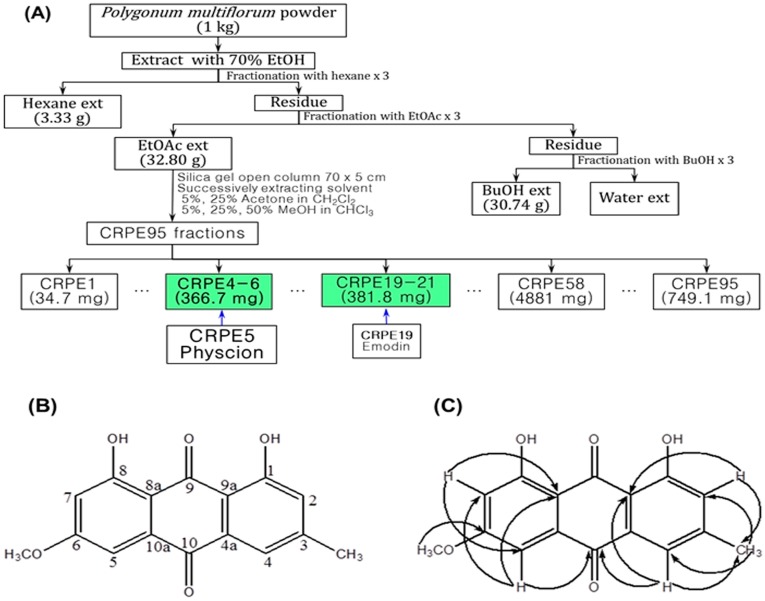
Purification chart (**A**); chemical structure (**B**); and key heteronuclear multiple bond correlation (HMBC) (**C**) of physcion from the ethyl acetate (EtOAc) extract of *Polygonum multiflorum* by silica gel column chromatography. CRPE is code name indicating ethyl acetate extract from *Polygonum multiflorum*. Physcion (CRPE5) and emodin (CRPE19) were finally isolated from CRPE4-6 and CRPE19-21 fractions, respectively.

After extraction of the dried roots of *P. multiflorum* with ethyl acetate and evaporation of the solvent, the remaining residue was chromatographed over silica gel. Among the compounds isolated by an extensive separation process, a compound displaying induction activity for hST8Sia VI gene expression in SK-N-BE(2)-C cells was selected as the active compound. This compound was clarified to be physcion (Figure 1) by gas chromatography-mass spectrometry (GC-MS) and ^1^H- and ^13^C-nuclear magnetic resonance (NMR). GC-MS analysis showed that the isolated physcion had a purity of more than 97%.

### 2.2. Effect of Physcion on Gene Expression of hST8Sia VI and Cell Proliferation

**Figure 2:**
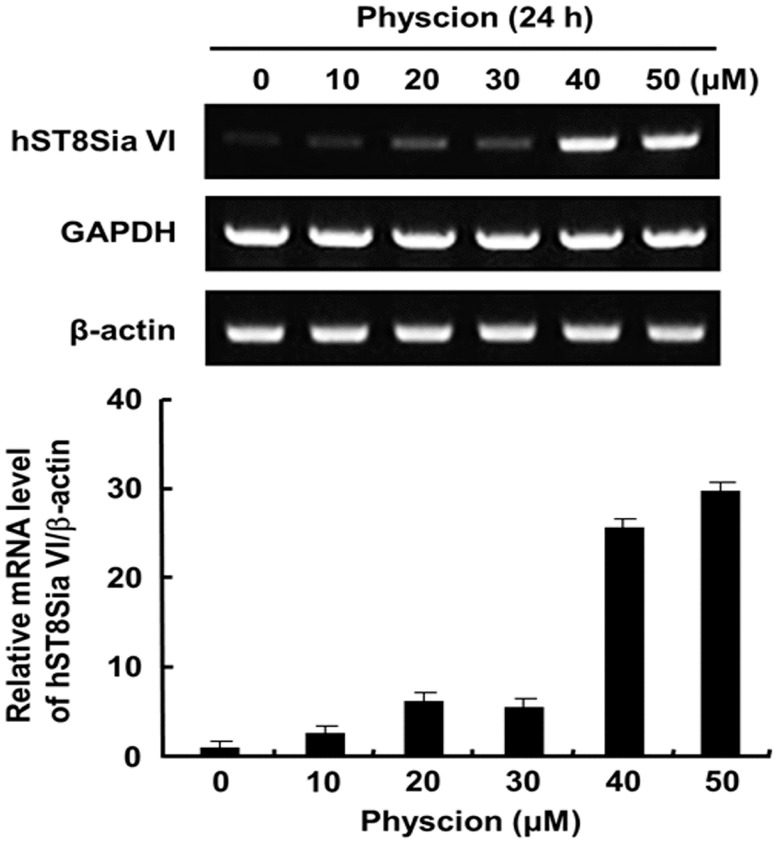
Effect of physcion on hST8Sia VI mRNA level in SK-N-BE(2)-C cells. After 24 h treatment with various concentration of physcion in the **panel up**, total RNA from SK-N-BE(2)-C cells was isolated. RT-PCR was used to detect hST8Sia VI mRNA level. In the **panel below**, the levels of each RT-PCR product were analyzed by densitometry and normalized by β-actin mRNA level as a control. Data reveal the relative values ± SD of three independent experiments.

**Figure 3:**
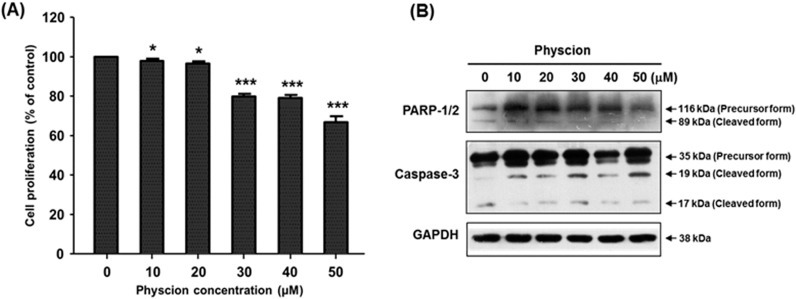
Effect of physcion on the viability and apoptosis of SK-N-BE(2)-C cells. (**A**) The cytotoxic effects of physcion on SK-N-BE(2)-C cells were examined using MTT assay. After 24 h incubation at various concentration of physcion, and cell viability was analyzed at 540 nm using an enzyme-linked immunosorbent assay (ELISA) plate reader (Bio-Rad, Hercules, CA, USA). The results represent the means ± SEM of three independent experiments. * *p* < 0.05 (compared with control); *** *p* < 0.001; (**B**) PARP-1/2 and caspase-3 were checked by Western blotting analysis.

To investigate the effect of physcion on the human sialyltransferase gene expression in SK-N-BE(2)-C cells, mRNA levels of nineteen human sialyltransferases were checked by RT-PCR using total RNAs obtained from cells after 24 h treatment with 40 μM physcion. The result showed that with the exception of hST8Sia VI, the expression levels of eighteen human sialyltransferase genes were not affected by physcion treatment when compared to the control without physcion (Figure S1). Next, we treated the cells for varying periods of time with varying doses of physcion, and checked the transcript level of hST8Sia VI. As shown in Figure 2, hST8Sia VI mRNA levels were significantly increased in SK-N-BE(2)-C cells treated with 40 μM physcion for 24 h. This result clearly revealed that the gene expression of hST8Sia VI was induced by physcion. Next, we checked the cytotoxicity of physcion in SK-N-BE(2)-C cells using MTT assay. As shown in Figure 3A, 24 h incubation with 20 μM physcion did not alter the viability of the cells, whereas treatment with 40 μM physcion reduced the cell viability by about 20% when compared to the control, indicating that physcion has a slight cytotoxic effect at 40 μM. To assess the effect of physcion on apoptosis in SK-N-BE(2)-C cells, the samples obtained after treatment with various concentrations of physcion for 24 h were subjected to SDS-PAGE. Subsequently, caspase-3 activation and poly (ADP-ribose) polymerase (PARP) cleavage were investigated by immunoblot analysis with the corresponding antibodies. As shown in Figure 3B, the distinct increase of caspase-3 and PARP cleavages, typical apoptosis markers, was not observed after treatment with 50 μM physcion for 24 h, suggesting that physcion might not induce apoptosis at the highest tested concentration (50 μM) in SK-N-BE(2)-C cells.

### 2.3. Isolation and Sequence Analysis of the 5′-Flanking Region of the hST8Sia VI Gene

Using the NCBI database, sequence comparison of the cDNA and the genomic DNA of the hST8Sia VI gene showed that the predicted transcription start site was located at 136 bp upstream of the ATG start codon. Based on this result, a 2660 bp region upstream of the transcription start site for the hST8Sia VI gene was obtained by LA-PCR and sequenced. By using a transcription factor binding site-prediction program, putative transcription factor binding sites (TFBS) within this region were screened and 52 TFBS were predicted (similarity margin ≥95%).

### 2.4. Promoter Analysis of the 5′-Flanking Region of the hST8Sia VI Gene in Physcion-Induced SK-N-BE(2)-C Cells

**Figure 4:**
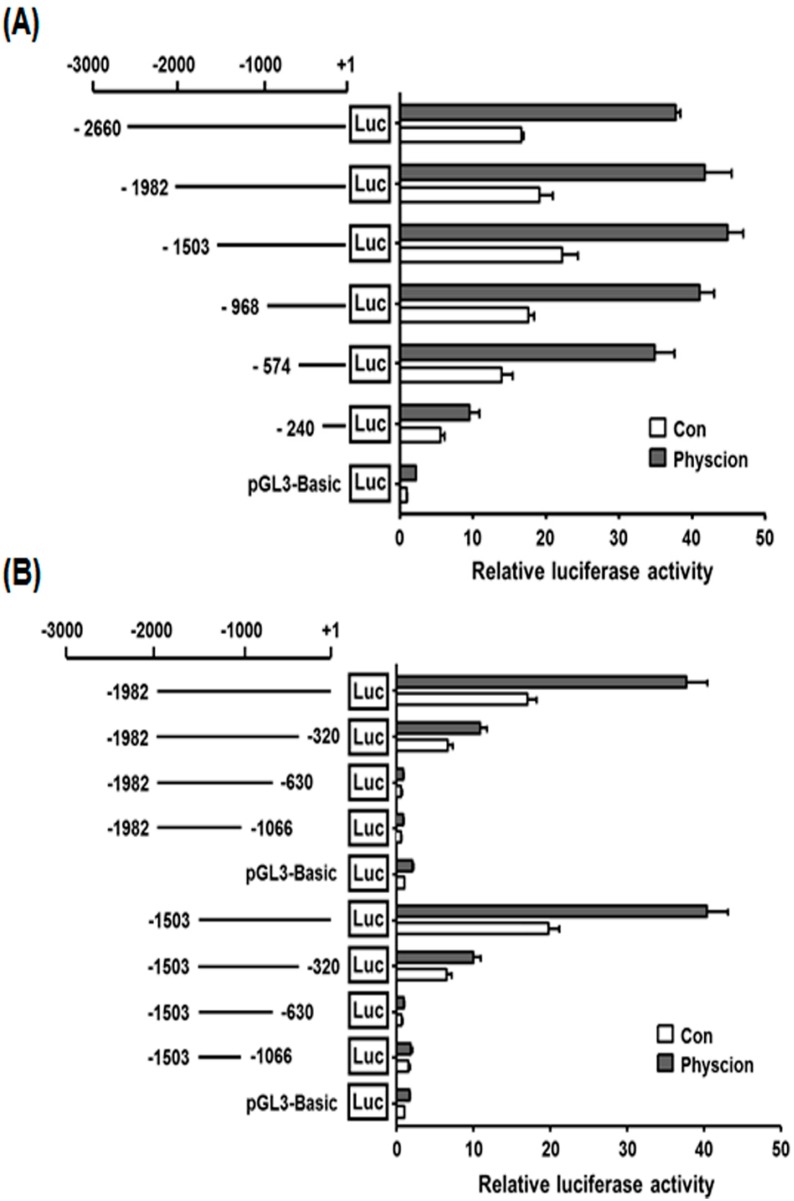
Deletion analysis of hST8Sia VI promoter in SK-N-BE(2)-C cells with physcion treatment. Reporter plasmids contain progressive deletions from the 5′ end (**A**) and the 3′ end (**B**) of the hST8Sia VI gene promoter. After co-transfection of each construct into SK-N-BE(2)-C cells with pRL-TK, relative firefly luciferase (Luc) activity was assessed. The pRL-TK-derived *Renilla* luciferase activity was used to normalize all firefly luciferase activity. Data are the means ± SD of triplicate measurements and are representative of three independent experiments.

To assess whether or not the 5′-flanking region of the hST8Sia VI gene includes a physcion-responsive promoter, six kinds of luciferase reporter plasmids (pGL3-2660, pGL3-1982, pGL3-1503, pGL3-968, pGL3-574, and pGL3-240) were constructed by inserting truncated fragments of the 5′-flanking region of the hST8Sia VI gene into the pGL3-Basic vector for analysis of luciferase activity. To identify physcion-responsive promoter activity, we transfected pGL3-Basic plasmid as a negative control along with the six reporter plasmids into physcion-untreated SK-N-BE(2)-C cells and checked the hST8Sia VI promoter activity by physcion induction. As shown in Figure 4A, all deletion plasmid constructs containing 5′-flanking regions between −2660 and −574 exhibited about two-fold higher promoter activity in physcion-treated cells than in physcion-untreated cells. However, when the 5′-flanking region was deleted up to −240, promoter activity by physcion induction was remarkably decreased. These results clearly indicate that the region between nucleotides −574 and −240 was crucial for the transcriptional activity of the hST8Sia VI gene in physcion-stimulated SK-N-BE(2)-C cells and that physcion-responsive elements exists within nucleotide −574 to −240 in the hST8Sia VI promoter.

Based on this result, to identify the minimal physcion-responsive region controlling the maximal promoter activity of the hST8Sia VI gene in SK-N-BE(2)-C cells, we constructed two different kinds of reporter plasmids (pGL3-1982/-320 to pGL3-1982/-1066 and pGL3-1503/-320 to pGL3-1503/-1066) containing progressive deletions from the 3′ end of the hST8Sia VI gene promoter and transfected them into physcion-untreated SK-N-BE(2)-C cells, then checked for promoter activity by physcion stimulation. As shown in Figure 4B, deletion in the region from +1 to −320 remarkably reduced the promoter activity in SK-N-BE(2)-C cells with or without physcion induction. Collectively, these results apparently suggest that the physcion-responsive core promoter region for the transcription of the hST8Sia VI gene in SK-N-BE(2)-C cells is located between −320 and −240.

### 2.5. Identification of Physcion-Responsive Element in the Functional −320/−240 Region of hST8Sia VI Promoter

**Figure 5:**
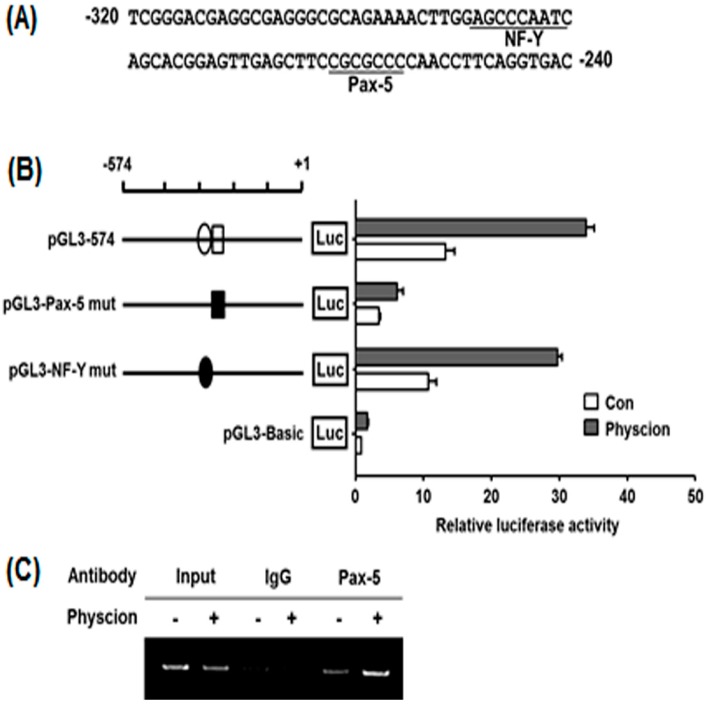
Promoter mutation assay for the transcription factor-binding sites in the nucleotide −320 to −240 region of the hST8Sia VI gene in physcion-induced SK-N-BE(2)-C cells. (**A**) Nucleotide sequences of the promoter region from −320 to −240 are shown; (**B**) After co-transfection of each construct into SK-N-BE(2)-C cells with pRL-TK, relative firefly luciferase activity was assessed. The *Renilla* luciferase activity derived from pRL-TK was used to normalize all firefly luciferase activity. Data are the means ± SD of triplicate measurements and are representative of three independent experiments. The circle and square indicate NF-Y and Pax-5 binding sites, respectively. The white and black colors indicate wild-type and mutant forms of these sites, respectively; (**C**) ChIP assay was conducted with primers amplifying the −379 and −131 region of the hST8Sia VI promoter and DNA isolated from chromatin immunoprecipitated with either anti-Pax-5 antibody or control IgG from SK-N-BE(2)-C cells treated for 24 h with or without 40 μM physcion.

To identify physcion-responsive elements in the nucleotide −320 to −240 region of the hST8Sia VI gene, we searched for TFBS within the nucleotide −320 to −240 region using bioinformatics analysis. Sequence analysis using the PROMO program [20,21] showed potential NF-Y (−289 to −282) and Pax-5 (−262 to −256) binding sites in this region (Figure 5A). To define whether these binding sites play a critical role in hST8sia VI expression by physcion induction in SK-N-BE(2)-C cells, two mutant plasmids (pGL3-Pax-5 mut and pGL3-NF-Y mut) were constructed (Figure 5B) and transfected into physcion-uninduced SK-N-BE(2)-C cells. Afterward, luciferase assays by physcion induction were performed. As shown in Figure 5B, the transcriptional activity of pGL3-Pax-5mut was markedly decreased to more than five-fold that of pGL3-574, whereas the activity of pGL3-NF-Ymut was slightly reduced. This result indicates that the Pax-5 binding site located at position −262 to −256 is crucial for the physcion-induced expression of the hST8Sia VI gene, and suggests that Pax-5 binding to this site is essential for the induction of hST8Sia VI gene expression by physcion stimulation. Next, we further evaluated whether Pax-5 can bind to this binding site in the hST8SIa VI promoter using chromatin immunoprecipitation (ChIP) assay, which examined the in vivo association of Pax-5 with the promoter region between positions −262 and −256 of the hST8STSia VI gene in physcion-treated SK-N-BE(2)-C cells. The specific PCR products obtained with input DNA are shown in Figure 5C. Although Pax-5-specific amplification by PCR was observed in physcion-untreated SK-N-BE(2)-C cells, physcion treatment resulted in a significant increase in Pax-5 binding in SK-N-BE(2)-C cells. On the other hand, a control assay using IgG antibody did not generate the PCR product in the absence or presence of physcion. These results indicate that the hST8Sia VI gene expression in physcion-induced SK-N-BE(2)-C cells is upregulated by the direct binding of Pax-5 to its site on the hST8Sia VI promoter region.

### 2.6. Transcriptional Activation of hST8Sia VI via ERK and p-38 MAPK Pathways in Physcion-Stimulated SK-N-BE(2)-C Cells

**Figure 6:**
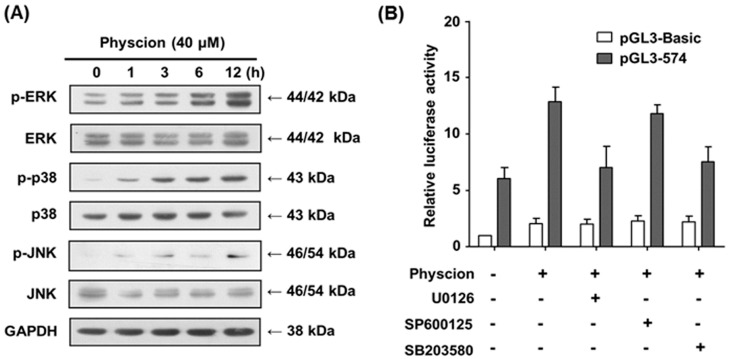
Physcion induced transcriptional activation of hST8Sia VI through ERK/p-38 MAPK signal pathways in SK-N-BE(2)-C cells. For representative immunoblot analysis of p-ERK, ERK, p-JNK, JNK, p-p38 and p38 protein expression levels, cells were treated with 40 μM physcion for different lengths of time (**A**). pGL3-574 or pGL3-Basic was co-transfected into SK-N-BE(2)-C cells with pRL-TK. The transfected cells were incubated for 24 h in the presence of 40 μM physcion with different inhibitors (10 μM U0126, 10 μM SP600125, and 20 μM SB203580); The *Renilla* luciferase activity derived from pRL-TK was used to normalize all firefly luciferase activity. Data are the means ± SD of triplicate measurements and are representative of three independent experiments (**B**). +, treatment; -, untreatment.

To uncover the mechanism underlying the transcriptional activation of hST8Sia VI gene by physcion induction, we examined the MAPK signal pathway in response to physcion stimulation in SK-N-BE(2)-C cells. As shown in Figure 6A, the expression of phosphorylated ERK and p38 MAPK were clearly enhanced in a time-dependent fashion in physcion-induced SK-N-BE(2)-C cells, whereas JNK phosphorylation was slightly increased by physcion induction. This result suggests that ERK and p38 MAPK, including JNK, are activated by physcion induction in SK-N-BE(2)-C cells.

To further assess whether physcion-stimulated transcriptional activity of a pGL3-574 promoter is induced via ERK and p38 MAPK signal pathways, we checked the promoter activity of pGL3-574 by physcion induction in SK-N-BE(2)-C cells pre-treated with MAPK inhibitors. As shown in Figure 6B, the promoter activity of pGL3-574 in SK-N-BE(2)-C cells induced by physcion was significantly decreased by ERK inhibitor U0126 and p38 MAPK inhibitor SB203580, but not by JNK inhibitor SP600125. Taken together, these results suggest that transcriptional activation of hST8Sia VI in physcion-stimulated SK-N-BE(2)-C cells is regulated through ERK and p38 MAPK pathways.

## 3. Discussion

It was previously reported that hST8Sia VI acting on Neu5Acα2-3Galβ1-3GalNAcα1-*O*-Ser/Thr or Neu5Acα2-6GalNAcα1-*O*-Ser/Thr in *O*-glycans was highly expressed in MCF-7 breast cancer cell line, whereas its expression was very low in MDA-MB-231 and T47-D breast cancer cell lines and in HT-29 and Caco-2 colon cancer cell lines [22]. However, nothing is known about the transcriptional regulation hST8Sia VI gene. This study presents the first report on the transcriptional regulation hST8Sia VI gene. Here, we have demonstrated for the first time that hST8Sia VI gene expression was upregulated in SK-N-BE(2)-C cells stimulated with physcion and that the Pax-5 binding site at position −262 to −256 plays a pivotal role in the transcriptional activation of hST8Sia VI by physcion induction.

Physcion, an anthraquinone derivative, has been reported to have a variety of pharmacological effects such as hepatoprotective [14], anti-microbial [15], anti-inflammatory [16], anti-cancer [13,17,18], and anti-metastatic activities [19]. However, the effect of physcion on gene expression, including sialyltransferase, has not been studied before. This study also represents the first report on the effect of physcion on gene expression and the underlying mechanism. Our results indicated that physcion treatment in SK-N-BE(2)-C cells enhanced hST8Sia VI mRNA levels.

In this study, we isolated the 2.6 kb 5′-flanking region upstream of the transcription start site of the hST8Sia VI gene. While a number of putative regulatory *cis*-acting elements were found in this region, typical TATA and CCAAT boxes were not existed. Moreover, we demonstrated that a functional promoter in response to physcion was located at the 5′-flanking region (pGL3-2660) of the hST8Sia VI gene in SK-N-BE(2)-C cells. Our present result clarified that the region between −574 and −240 is the core promoter essential for transcriptional activation of hST8Sia VI gene in physcion-induced SK-N-BE(2)-C cells, as evidenced by deletion analysis. This region contains NF-Y (−289 to −282) and Pax-5 (−262 to −256) binding sites. By site-directed mutagenesis experiments and ChIP assay, we also verified that the Pax-5 binding site in this region plays a pivotal role in the transcriptional activation of the hST8Sia VI gene in physcion-stimulated SK-N-BE(2)-C cells.

Pax-5 is well known as the B cell-specific transcription factor that plays a crucial role in the development and malignancy of B cells [23,24]. Pax5 regulates B lymphopoiesis through the activation of B cell-specific gene expression and concurrent repression of the expression of non-B cell genes [23]. It was documented that Pax-5 is mainly expressed at the midbrain-hindbrain boundary during mouse development, suggesting that Pax-5 is associated with the development of the central nervous system [25]. Pax-5 expression has also been reported in various cancer cells, including medulloblastoma [26], lymphoma [27], small-cell lung carcinoma [28], and neuroblastoma [29,30]. Previous studies have shown that SK-N-BE(2)-C cells expressed lower levels of Pax-5 [29], and downregulation of Pax-5 in SK-N-BE(2)-C cells resulted in significant reduction in the cell proliferation rate [30].

It was recently reported that stimulation of mouse primary B cells by lipopolysaccharide (LPS)-induced T-independent (TI) or IL4/IL5/CD40L-induced T-dependent (TD) pathway resulted in remarkable elevation of the mouse ST8Sia VI gene expression in mature and plasma cell populations as compared with ST8Sia II and ST8Sia IV [31]. This finding suggests transcriptional upregulation of ST8Sia VI during the differentiation of plasma cells by TI and TD stimulation. Considering that Pax-5 plays a role in mature B-cell differentiation [23], this finding also suggests the possibility that Pax-5 may be related to the transcriptional upregulation of ST8Sia VI, although it remains to be elucidated whether a Pax-5 binding site exists in the core region of the mouse ST8Sia VI promoter.

Our data show that ERK, JNK and p38 MAPK activation were induced by physcion in SK-N-BE(2)-C cells, although physcion-mediated MAPK signaling pathways are not known at present. The present result also indicates that transcriptional activation of hST8Sia VI is mediated through ERK and p38 MAPK pathways induced by physcion in SK-N-BE(2)-C cells, as demonstrated by luciferase assay using cells treated with chemical inhibitors that selectively or specifically block these pathways.

## 4. Experimental Section

### 4.1. Extraction, Isolation and Structure Determination of Physcion

Extraction and isolation of physcion from the dried root of *Polygonum multiflorum* were carried out by the same procedures as those described previously [32]. The structure of the purified physcion by ^1^H and ^13^C NMR spectroscopy with a Varian Spectrometer (Palo Alto, CA, USA) at 600 MHz with CDCl_3_ was determined. The gas chromatography-mass spectrometry (GC-MS) profiles of the purified physcion were recorded as described previously [32]. ^1^H NMR and ^13^C NMR results were compared with the data presented in the references [33,34].

### 4.2. Cell Cultures

Human neuroblastoma SK-N-BE(2)-C cells were obtained from American Type Culture Collection (Manassas, VA, USA) and cultured in Dulbecco’s modified Eagle’s medium (DMEM) supplemented with 100 μg/mL of streptomycin, 100 U/mL of penicillin, and 10% fetal bovine serum (WelGENE Co., Daegu, Korea) at 37 °C in a 5% CO_2_ incubator.

### 4.3. Cell Viability Assay

Cell viability assay was performed as described previously [7,8,9,10,11]. The amount of formazan produced was quantified by measuring the absorbance at 490 nm with an ELISA plate reader (Bio-Rad, Hercules, CA, USA).

### 4.4. Reverse Transcription-Polymerase Chain Reaction (RT-PCR)

Total RNA isolation and first-strand cDNA synthesis were performed as described previously [35]. PCR amplification was performed with a PC-818A Program Temp Control System (Astec, Fukuoka, Japan), with 1 cycle for 5 min at 95 °C and 30 cycles consisting of denaturation at 95 °C for 40 s, annealing at 60 °C for 40 s, and extension for 45 s at 72 °C, followed by incubation at 72 °C for 5 min. PCR products were analyzed by 1% agarose gel electrophoresis. Quantitation of the intensity of the amplified bands was performed using a Scion Image Instrument (Scion Corp.; Frederick, MD, USA).

### 4.5. Cloning of the 5′-Flanking Region of the hST8Sia VI Gene and Bioinformatics Analysis

Using the sequence information for hST8Sia VI cDNA (GenBank accession number BC137102) and Homo sapiens chromosome 10, GRCh38.p2 Primary Assembly (GenBank accession number NC_000010 GPC_000001302) from the National Center for Biotechnology Information (NCBI), a 2660 bp fragment from the 5′-flanking sequence of the hST8Sia VI gene was amplified by long and accurate PCR (LA-PCR) with LA-*Taq* polymerase (Takara Bio, Shiga, Japan). LA-PCR was performed with the sense primer P-2660S containing a *Kpn*I site and the antisense primer P-2660A containing a *Xho*I site (Table 1). Human genomic DNA as a template was isolated from SK-N-BE(2)-C cells. The LA-PCR condition was 1 cycle for 1 min at 95 °C and then 30 cycles of 98 °C for 20 s and 68 °C for 3 min, with a final extension for 10 min at 72 °C. The amplified PCR products were subcloned into pGEM-T Easy vector (Promega, Madison, WI, USA) to produce pGST8Sia VI. PCR products were sequenced in both directions by cloning convenient restriction fragments into the pUC118/9 vector. Putative binding sites for the transcription factors of the 5′-flanking region were analyzed using ALGGEN-PROMO search algorithm (http://alggen.lsi.upc.es/) based on TRANSFAC version 8.3 [20,21] with a maximum matrix dissimilarity rate of 5.

### 4.6. Construction of Luciferase Reporter Plasmids and Mutagenesis

To determine the physcion-activated minimal promoter region of the hST8Sia VI gene, three kinds of luciferase reporter plasmids (pGL3-240 to pGL3-2660, pGL3-1982/-320 to pGL3-1982/-1066, and pGL3-1503/-320 to pGL3-1503/-1066), were constructed by LA-PCR with sense and antisense primers containing *Kpn*I and *Xho*I sites, respectively (Table 1), using the pGST8Sia VI described above as the template. The PCR fragments were subcloned into pGEM-T Easy vector and sequenced. Each fragment produced by digestion with *Kpn*I and *Xho*I was introduced into the corresponding sites of the pGL3-Basic vector used as a negative control. Mutations with base substitution and deletion at the Pax-5 and NF-Y binding sites, respectively, were generated using a QuikChange^®^ II XL site-directed mutagenesis kit (Stratagene, La Jolla, CA, USA) according to the manufacturer’s protocol using oligonucleotide primers (Table 1). The presence of mutation was verified by DNA sequencing.

### 4.7. Transfection and Luciferase Assay

Transient transfection and luciferase assay were carried out as described previously [7,8,9], 0.5 μg of luciferase reporter plasmid and 50 ng of pRL-TK as the control *Renilla* luciferase vector (PRomega; Madison, WI, USA) were co-transfected into cells using 1 μL Lipofectamine 2000 (Invitrogen, Carlsbad, CA, USA). After 4 h culture, the transfection medium was replaced by normal medium without physcion and incubated for 7 h. Then, the medium was changed to medium containing 40 μM physcion (Sigma, St. Louis, MO, USA) and incubated for 24 h. Cells were harvested and assayed using the Dual-Luciferase Reporter Assay System (Promega, Madison, WI, USA) and a GloMax™ 20/20 luminometer (Promega).

### 4.8. Western Blot Analysis

Western blot analysis was conducted as described previously [35]. Thirty-five microgram samples of total cell lysates prepared using RIPA buffer were subjected to SDS-PAGE and transferred to PVDF membranes. The blotted membranes were incubated with primary and secondary antibodies. Blots were detected using the ECL chemiluminescence system (GE Healthcare, Piscataway, NJ, USA). The following primary antibodies were used: p-ERK, ERK (Santa Cruz, CA, USA), p-p38, p38 (Cell Signaling Technology, Beverly, MA, USA) and GAPDH (Millipore, Billerica, MA, USA). Horseradish peroxidase (HRP)-conjugated secondary antibodies were purchased from Enzo Life Science (Farmingdale, NY, USA).

### 4.9. Chromatin Immunoprecipitation (ChIP) Assay

ChIP assay was performed using the ChIP kit (Upstate Biotechonology, Lake Placid, NY, USA) according to the manufacture’s protocol. Formaldehyde cross-linking of cells and shearing of chromatin were carried out as described previously [9,35]. Immunoprecipitation was conducted with Pax-5 (A-11, Santa Cruz, CA, USA) and IgG antibodies (Sigma; St. Louis, MO, USA). PCR analysis was conducted with primers flanking the Pax-5 binding site on the hST8Sia VI promoter (Table 1) and the purified ChIP DNA or input DNA. The PCR condition was 1 cycle at 94 °C for 3 min, followed by 32 cycles with a denaturing step at 94 °C for 20 s, an annealing step of 30 s at specific annealing temperatures for 59 °C, an elongation step at 72 °C for 30 s and a final extension step at 72 °C for 2 min.

## 5. Conclusions

In the present study, we have demonstrated for the first time that hST8Sia VI gene expression was upregulated in SK-N-BE(2)-C cells stimulated with physcion and that the region between −320 and −240, which contains putative binding sites for Pax-5 and NF-Y in the hST8Sia VI promoter, acts as the major promoter for transcriptional activation of hST8Sia VI in physcion-induced SK-N-BE(2)-C cells. Furthermore, the Pax-5 binding site at position −262 to −256 plays a crucial role in the transcriptional activation of hST8Sia VI by physcion induction, as demonstrated by mutagenesis and ChIP assay. Our data suggest that physcion-induced transcriptional activation of hST8Sia VI is mediated through ERK and p38 MAPK pathways in SK-N-BE(2)-C cells.

## Figures and Tables

**Table 1:** Primer sequences used for reverse transcription-polymerase chain reaction (RT-PCR) and deletion constructs, site-directed mutagenesis and chromatin immunoprecipitation (ChIP).

Primer	Sequence	Strand	Purpose
hST8Sia VI	5′-CCCTATTTCTGGAGGACATTGCAACCTA-3′	Sense	RT-PCR
hST8Sia VI	5′-GTTGGAGGATCTGGCTGTATTCTTTG-3′	Antisense	RT-PCR
β-actin	5′-CAAGAGATGGCCACGGCTGCT-3′	Sense	RT-PCR
β-actin	5′-TCCTTCTGCATCCTGTCGGCA-3′	Antisense	RT-PCR
GAPDH	5′-AGCCTCAAGATCATCAGCAATGTCCT-3′	Sense	RT-PCR
GAPDH	5′-AAATTCGTTGTCATACCAGGAAATGAG-3′	Antisense	RT-PCR
P-2660	5′-ATGGTACCCTTCTGCTGTTGCCTTGAGCCCAGC-3′	Sense	Deletion
P-2660	5′-ATCTCGAGACAGCGTTCACAGGCGGCAGCGAG-3′	Antisense	Deletion
P-1982	5′-ATGGTACCGGCTGTCTGGCCTGGTTGCTCCCA-3′	Sense	Deletion
P-1503	5′-ATGGTACCAAGGATACCATAGGCTGGGTGACCG-3′	Sense	Deletion
P-968	5′-ATGGTACCAGGCTGCCTTGTGGGGCCTGGTATA-3′	Sense	Deletion
P-574	5′-ATGGTACCGCCCCTCATACCAGTTCGCTGTCCC-3′	Sense	Deletion
P-240	5′-ATGGTACCCGCGCGGCGGCGGCGGCAGCAGC-3′	Sense	Deletion
P-320A	5′-ATCTCGAGTTCTGCGCCCTCGCCTCGTCCCGA-3′	Antisense	Deletion
P-630A	5′-ATCTCGAGCCTGGAGACCCGTTTAGCCCCTG-3′	Antisense	Deletion
P-1066A	5′-ATCTCGAGGGGTGGACCTCATGGACCTCCTC-3′	Antisense	Deletion
Pax-5 Mut	5′-GGAGTTGAGCTTC***CGCATTC*C**AACCTTCAGGTGACC-3′	Sense	Mutagenesis
Pax-5 Mut	5′-GGTCACCTGAAGGTT**G*GAATGCG*G**AAGCTCAACTCC-3′	Antisense	Mutagenesis
NF-Y Mut	5′-GCAGAAAACTTGG***AGCAAT***CAGCACGGAGTTGAGC-3′	Sense	Mutagenesis
NF-Y Mut	5′-GCTCAACTCCGTGCTG*A**TTGCT***CCAAGTTTTCTGC-3′	Antisense	Mutagenesis
hST8Sia VI	5′-AGGCAGAGTTGTGGTGTGGC-3′	Sense	ChIP
hST8Sia VI	5′-TGGCAGATGACGATTCGCCGA-3′	Antisense	ChIP

Primers P-2660S to P-240S were used for construction of the deletion mutants. *Kpn*I and *Xho*I sites in sense and antisense primers, respectively, are underlined. The mutated nucleotides in the oligonucleotides for mutation are in boldface and italic type.

## Supplementary Materials

Supplementary materials can be found at http://www.mdpi.com/1422-0067/17/8/1246/s1.
