# Supplementary material for: Upregulation of Human ST8Sia VI (α2,8-Sialyltransferase) Gene Expression by Physcion in SK-N-BE(2)-C Human Neuroblastoma Cells
Source: Int J Mol Sci. 2016 Aug 2;17(8):1246. doi: 10.3390/ijms17081246 (PMC5000644; doi:10.3390/ijms17081246)
Supplement: Supplementary file 1 [file ijms-17-01246-s001.pdf]

Supplementary Materials: Upregulation of Human ST8Sia VI (α2,8-Sialyltransferase) Gene Expression by Physcion in SK-N-BE(2)-C Human Neuroblastoma Cells

Hyun-Kyoung Yoon, Hyun-Kyu An, Min Jung Ko, Kyoung-Sook Kim, Seo-Won Mun, Dong-Hyun Kim, Cheol Min Kim, Cheorl-Ho Kim, Young Whan Choi and Young-Choon Lee

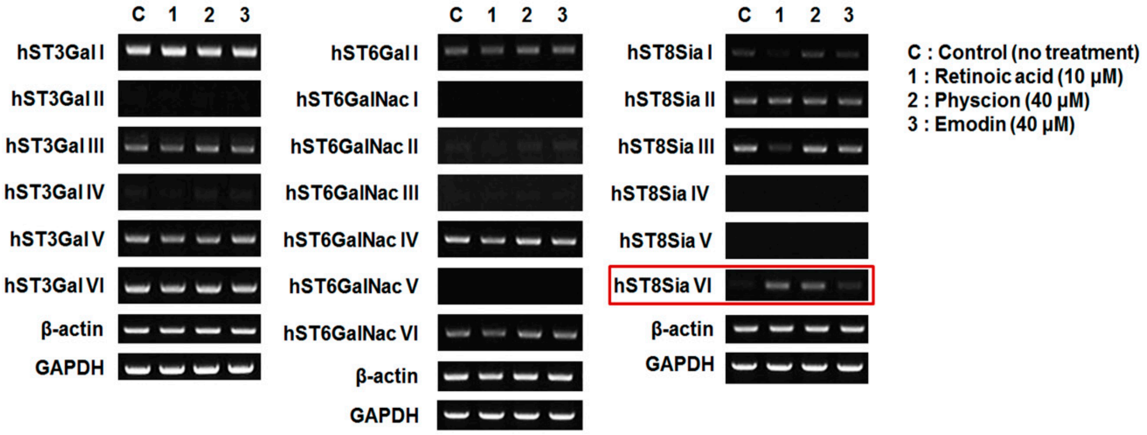

Figure S1. Effects of physcion on gene expression of human sialyltransferases in SK-N-BE(2)-C cells**.** SK-N-BE(2)-C cells were treated with retinoic acid (10 μM), physcion (40 μM), and emodin (40 μM) for 24 h. mRNA transcripts of human sialyltransferase were detected by RT-PCR. As the internal controls, parallel reactions were performed to measure levels of GAPDH and β-actin.

Table S1. Primer sequences used for reverse transcription-polymerase chain reaction (RT-PCR) of nineteen human sialyltransferase genes.

Primer	Sequence	Strand	Size (bp)
hST3Gal I	5′-CTGAAAGTGCTCACCTTCCTCGTGC-3′	Sense	
hST3Gal I	5′-ACAGCTCCTTGATGGTGTCATTCAA-3′	Antisense	337
hST3Gal II	5′-TACCTGGACTCAGGGGCCCTGGATG-3′	Sense	
hST3Gal II	5′-GGCACTGGTGGGGGTCCCGGAA-3′	Antisense	343
hST3Gal III	5′-GTATGATCGGTTGGGCTTC-3′	Sense	
hST3Gal III	5′-CGCTCGTACTGCTCAGG-3′	Antisense	520
hST3Gal IV	5′-GCTTTCACCTCTGTGCAG-3′	Sense	
hST3Gal IV	5′-CTTGTTGATGGCATCTCCC-3′	Antisense	240
hST3Gal V	5′-CCCTGCCATTCTGGGTACGAC-3′	Sense	
hST3Gal V	5′-CACGATCAATGCCTCCACTGAGATC-3′	Antisense	413
hST3Gal VI	5′-TTGGGAGAAGGACAACCTTC-3′	Sense	
hST3Gal VI	5′-CCAGGCAGCAACAGACAGTA-3′	Antisense	630
hST6Gal I	5′-TATCGTAAGCTGCACCCCAATC-3′	Sense	
hST6Gal I	5′-TTAGCAGTGAATGGTCCGGAAG-3′	Antisense	372
hST6GalNAc I	5′-GCAACCACAGCCAAGACGCTCATTCCC-3′	Sense	
hST6GalNAc I	5′-TGTCACGACCTTCTGCACCAAGGAGTAG-3′	Antisense	456
hST6GalNAc II	5′-AAGCTGCTACATCCGGACTTCA-3′	Sense	
hST6GalNAc II	5′-GGGACAGATCGTGGTTTGCATA-3′	Antisense	247
hST6GalNAc III	5′-CTGTGATTGCTGTGAGCTTCATAG-3′	Sense	
hST6GalNAc III	5′-AAGAGGAACGCTGGTATGGGACACA-3′	Antisense	380
hST6GalNAc IV	5′-CGGCTCGTGCTCATCATCCTGTGCT-3′	Sense	
hST6GalNAc IV	5′-GTGAGCTGCAGCAGCGTGCGGTAGG-3′	Antisense	503
hST6GalNAc V	5′-ATGAAGACCCTGATGCGCCATGGTC-3′	Sense	
hST6GalNAc V	5′-CTTGTGGCGAGTAATCATGAAGGCC-3′	Antisense	624
hST6GalNAc VI	5′-ATGAGTAGCAACAAAGAGCAGCGGT-3′	Sense	
hST6GalNAc VI	5′-AACACCAGGCCCGCTCGCTGGATCA-3′	Antisense	518
hST8Sia I	5′-TGTGGTCCAGAAAGACATTTGTGGACA-3′	Sense	
hST8Sia I	5′-TGGAGTGAGGTATCTTCACATGGGTCC-3′	Antisense	460
hST8Sia II	5′-ACCATGAATGTGTCCCAGAACCTCTA-3′	Sense	
hST8Sia II	5′-AGGTAGATTTGTTTGCAGAAACGTGTG-3′	Antisense	542
hST8Sia III	5′-GCTTGACGGGGCCATTCTTTGG-3′	Sense	
hST8Sia III	5′-GCTTGGTGAGCCCTTCCCCA-3′	Antisense	410
hST8Sia IV	5′-GCCACAGAAGACCCCAGTGA-3′	Sense	
hST8Sia IV	5′-CACTGACACATCTCGTTCTGCA-3′	Antisense	450
hST8Sia V	5′-ACCGGGATTTGTTGGGGAGCCGA-3′	Sense	
hST8Sia V	5′-GATGCCACTGGTGTCCACCTCA-3′	Antisense	392
hST8Sia VI	5′-CCCTATTTCTGGAGGACATTGCAACCTA-3′	Sense	
hST8Sia VI	5′-GTTGGAGGATCTGGCTGTATTCTTTG-3′	Antisense	390
β-actin	5′-CAAGAGATGGCCACGGCTGCT-3′	Sense	
β-actin	5′-TCCTTCTGCATCCTGTCGGCA-3′	Antisense	247
GAPDH	5′-AGCCTCAAGATCATCAGCAATGTCCT-3′	Sense	
GAPDH	5′-AAATTCGTTGTCATACCAGGAAATGAG-3′	Antisense	528

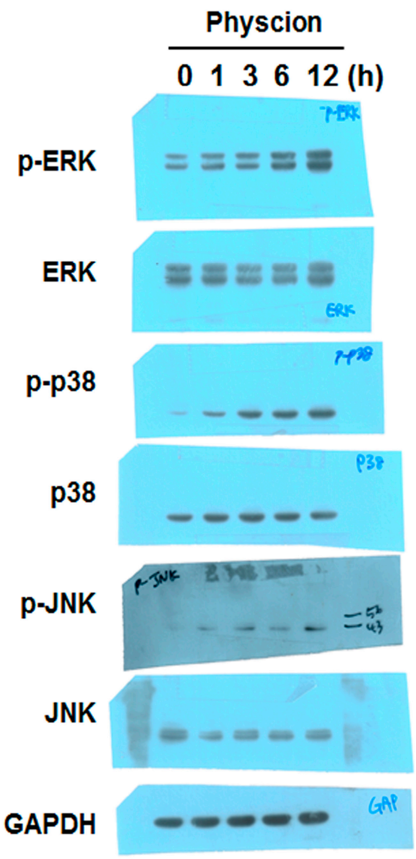

Figure S2. Raw data of Figure 6A.
